# Association Between Changes in Racial Residential and School Segregation and Trends in Racial Health Disparities, 2000–2020: A Life Course Perspective

**DOI:** 10.1007/s40615-024-01960-y

**Published:** 2024-02-29

**Authors:** Michael Siegel, Vanessa Nicholson-Robinson

**Affiliations:** Department of Public Health and Community Medicine, Tufts University School of Medicine, 136 Harrison Avenue, Boston, MA 02111 USA

**Keywords:** Racial health disparities, Structural racism, African Americans, Mortality rates, Life expectancy, Infant mortality, School racial segregation, Residential racial segregation

## Abstract

**Introduction:**

Most studies of the relationship between racial segregation and racial health disparities have focused on residential segregation. School-based racial segregation is an additional form of segregation that may be associated with racial disparities in health. This study examines the relationship between both residential segregation and school segregation and racial health disparities among non-Hispanic Black compared to non-Hispanic White persons at the county level in the United States. It also examines the relationship between changes in residential and school segregation and subsequent trajectories in a variety of racial health disparities across the life course.

**Methods:**

Using the CDC WONDER Multiple Case of Death database, we derived an annual estimate of race-specific death rates and rate ratios for each county during the period 2000–2020. We then examined the relationship between baseline levels of residential and school segregation in 1991 as well as changes between 1991–2000 and the trajectories of the observed racial health disparities between 2000 and 2020. We used latent trajectory analysis to identify counties with similar patterns of residential and school segregation over time and to identify counties with similar trajectories in each racial health disparity. Outcomes included life expectancy, early mortality (prior to age 65), infant mortality, firearm homicide, total homicide, and teenage pregnancy rates.

**Results:**

During the period 1991–2020, racial residential segregation remained essentially unchanged among the 1051 counties in our sample; however, racial school segregation increased during this period. Increases in school segregation from 1991 to 2000 were associated with higher racial disparities in each of the health outcomes during the period 2000–2020 and with less progress in reducing these disparities.

**Conclusion:**

This paper provides new evidence that school segregation is an independent predictor of racial health disparities and that reducing school segregation—even in the face of high residential segregation—could have a long-term impact on reducing racial health disparities. Furthermore, it suggests that the health consequences of residential segregation have not been eliminated from our society but are now being exacerbated by a new factor: school-based segregation. Throughout this paper, changes in school-based segregation not only show up as a consistent significant predictor of greater racial disparities throughout the life course, but at times, an even stronger predictor of health inequity than residential segregation.

**Supplementary Information:**

The online version contains supplementary material available at 10.1007/s40615-024-01960-y.

## Introduction

Residential racial segregation has long been recognized as a fundamental cause of racial health disparities [1] and has been associated with a wide range of adverse health outcomes for Black Americans, including infant mortality [2–4], low birth weight [5, 6], hypertension [7, 8], child health [9, 10], overall health [11], firearm violence [12, 13], fatal police shootings[14], and COVID-19 infections and outcomes [15–17]. However, there is an additional form of segregation that may be associated with racial disparities in health: racial school segregation. Patterns of residential and school racial segregation may be quite different [18–24]; therefore, studying the influence of segregation on racial health disparities may be incomplete if it does not consider both residential and school segregation. Few, if any studies have jointly evaluated the impact of both residential and school-based racial segregation on the magnitude of racial health disparities or changes in levels of racial health disparities over time. Moreover, we are not aware of any papers that have examined whether changes in school segregation are related to trends in racial health disparities in subsequent years.

Although the literature linking racial residential segregation and racial health disparities is robust [25], few studies have examined the association between school-based racial segregation and racial health disparities. Most literature on the health effects of racial school segregation has focused on immediate or short-term behavioral outcomes, such as youth smoking and alcohol use [26, 27], youth obesity [28, 29], juvenile crime [24], and teen pregnancy [30, 31]. We are aware of only three studies that related school segregation to long-term health outcomes [32–34]. Shen et al. found an association between higher levels of school segregation and an increased rate of pre-term births among Black mothers [32]. Kim et al. reported a relationship between higher levels of school segregation and worsened long-term cardiovascular health among Black adults [33]. Hahn estimated the impact of school racial segregation on life expectancy among the Black population but did not actually measure life expectancy [34]. The study examined the impact of school segregation on graduation rates and then relied upon separate data that estimated the relationship between graduation rates and life expectancy.

This article addresses this gap in the literature by evaluating the independent association at the county level between two forms of racial separation (residential and school segregation) and changes in both forms of segregation on a number of racial health disparities across the life course. Conceptually, we are guided by the life course perspective, which demonstrates that “current health is shaped by earlier exposures (even decades before) to physical, environmental, and psychosocial factors” [35, p. S48]. Thus, we examine the relationship between residential and school segregation levels and trends between 1991 and 2000 and trajectories in a number of racial health disparities across the life course during the subsequent two decades (2000–2020). The racial health disparities examined span the life course from adolescence (teen pregnancy) to young adulthood (infant mortality and firearm/overall homicide) to middle and later adulthood (early mortality and life expectancy). We examine racial disparities between the non-Hispanic Black and non-Hispanic White populations, since these are the racial/ethnic groups between which the greatest health disparities exist [36].

There are strong conceptual and empirical reasons to believe that the advantages and disadvantages conveyed by school-based racial segregation will translate into disparities in health trajectories across the life span. Cumulative disadvantage theory predicts that differential exposure to resources or deprivation during the school-age years may result in increasing health inequities across the life course [36]. Walsemann et al. have demonstrated that educational advantages or disadvantages in youth influence an individual’s health trajectory through middle age and therefore may result in persistent health disparities in adulthood [37]. There is a body of evidence showing that disadvantages in childhood influence health inequality over the life course [38–43].

This paper has three major aims: (1) to identify trends in racial residential and school segregation at the county level during the period 1991–2020, (2) to examine the independent association between early life residential and school segregation (1991–2000) and trajectories in racial health disparities across the life course (2000–2020), and (3) to investigate whether changes in residential or school segregation (1991–2000) influence subsequent trajectories in racial health disparities (2000–2020).

## Methods

### Measures and Data Sources

#### Outcome Variables

##### Racial Disparities in Life Expectancy

The Institute for Health Metrics and Evaluation (IHME) has made publicly available annual estimates of life expectancy by race/ethnicity at the county level for the period 2000–2019 [44]. From these data, we extracted annual estimates of life expectancy at birth for the non-Hispanic Black and non-Hispanic White population in each county. The racial disparity was defined as the absolute difference between life expectancy for the White compared to the Black population (i.e., White life expectancy minus Black life expectancy). A full set of estimates was available for 1501 counties.

##### Racial Disparities in Early Mortality

We defined early mortality as death prior to the age of 65. We obtained annual age-adjusted early mortality rates for the non-Hispanic Black and non-Hispanic White population in each county for the years 2000–2020 using the CDC WONDER Multiple Cause of Death database [45], including deaths from all causes for decedents less than 65 years old. The racial disparity was defined as the mortality rate ratio, or the Black early mortality rate divided by the White early mortality rate. To protect privacy, the CDC does not report death counts of less than 10, and it warns that rates based on death counts of less than 20 are unreliable. Therefore, we only included counties in which there were at least 20 deaths for each race-year combination. A complete set of estimates was available for 538 counties.

##### Racial Disparities in Infant Mortality Rates

Infant mortality is defined as death occurring during the first year of life. We obtained annual infant mortality rates for the non-Hispanic Black and non-Hispanic White population in each county for the years 2001–2018 using the CDC WONDER Multiple Cause of Death database [45], including deaths from all causes for decedents less than 1 year of age. The racial disparity was defined as the mortality rate ratio, or the Black infant mortality rate divided by the White infant mortality rate. Following CDC recommendations, rates were only used if they were based on at least 20 deaths. To increase the county sample size, we derived 5-year moving averages of infant mortality rates. Thus, the infant mortality rate used for 2001 was the 5-year overall infant mortality rate for the years 1999 to 2003. A complete set of estimates was available for 199 counties.

##### Racial Disparities in Firearm Homicide Rates

We obtained annual total and firearm homicide rates for the non-Hispanic Black and non-Hispanic White population in each county for the years 2002–2017 using the CDC WONDER Multiple Cause of Death database [45], including deaths from homicide and legal intervention (i.e., killings by police). The racial disparity in total homicide was defined as the Black total homicide rate divided by the White total homicide rate. Similarly, the racial disparity in firearm homicide was defined as the Black firearm homicide rate divided by the White firearm homicide rate. Following CDC recommendations, rates were only used if they were based on at least 20 deaths. To increase the county sample size, we derived 7-year moving averages of total and firearm homicide rates. Thus, the homicide rate used for 2002 was the 7-year overall homicide rate for the years 1999 to 2005. A complete set of firearm homicide rate estimates was available for 104 counties, and a complete set of total homicide rate estimates was available for 183 counties.

##### Racial Disparities in Teen Birth Rates

We obtained data on the teen birth rate among the non-Hispanic Black and non-Hispanic White populations of each county for the period 2014–2020 from the County Health Rankings and Roadmaps 2023 database [46]. Teen birth rates were defined as the number of births per 1000 teens ages 15–19 for each racial/ethnic group. The racial disparity was defined as the non-Hispanic Black teen birth rate divided by the non-Hispanic White teen birth rate. Data were available for 1019 counties.

#### Predictor Variables: Racial Residential and Racial School Segregation

##### Measures of Racial Residential Segregation

For each county, we produced annual estimates of two common measures of Black-White racial segregation—the index of dissimilarity and the entropy index—using Census tract-level population data from the 1990, 2000, 2010, and 2020 Decennial Censuses [47, 48]. The Decennial Census data for 2000, 2010, and 2020 were downloaded directly from the US Census Bureau web site [47]. The data for 1990 were obtained from the Inter-university Consortium for Political and Social Research (ICPSR) [48]. To derive annual estimates for each measure, we linearly interpreted the data between 1990 and 2000, 2000 and 2010, and 2010 and 2020. Because residential segregation changes very slowly, it is reasonable to assume that there are no wide fluctuations that would render the linear interpolation inaccurate.

##### Index of Dissimilarity

A commonly used measure of racial segregation, the index of dissimilarity represents the proportion of people of one racial group that would have to move from a smaller level of geography to fully integrate a larger level of geography [49]. The scale runs from 0 to 100: 0 indicates full integration and 100 indicates complete segregation. We calculated the index of dissimilarity for the non-Hispanic White and non-Hispanic Black populations in each county using the Census tract as the lower level of geography and the county as the larger unit. The formula was:

$${\text{I}}=\frac{1}{2}{\sum }_{t=1}^{n}\left|\left({B}_{t}/{B}_{\text{C}}\right)-\left({W}_{t}/{W}_{\text{C}}\right)\right|$$

where ${B}_{t}$ is the number of Black people in Census tract *t*, ${B}_{\text{C}}$ is the number of Black people in the county as a whole, ${W}_{t}$ is the number of White people in Census tract *t*, ${W}_{\text{C}}$ is the number of White people in the county as a whole, and the sum is across all $n$ Census tracts within the county.

##### Entropy Index

The entropy index compares the proportion of each racial group in a lower geographical unit to its proportion in a higher geographical unit [50, 51]. Thus, the census tract was the lower unit, and the county was the higher unit. The entropy index, then, compared the proportion of non-Hispanic Black people in each census tract to the expected proportion based on the overall county proportion of non-Hispanic Black people and the same for the proportion of non-Hispanic White people in each census tract. The entropy index runs from 0 to 100, with 0 indicating full integration and 100 indicating complete segregation. For a given census tract with $n$ racial groups, its entropy (E_*t*_) is defined as:

$${\sum }_{r\text{=}1}^{n}{P}_{rt}\left(1/{P}_{rt}\right) \, ,$$

where ${{\text{P}}}_{{\text{rt}}}$ is the proportion of tract ${\text{t}}$’s population comprised of racial group ${\text{r}}$.

The entropy for a county with $n$ racial groups (E_*C*_) is defined similarly as:

$${\sum }_{r\text{=}1}^{n}{{\text{P}}}_{{\text{r}}{\text{C}}}\left(1/{{\text{P}}}_{{\text{r}}{\text{C}}}\right),$$

where ${P}_{r{\text{C}}}$ is the proportion of the county’s population comprised of racial group $r$.

The entropy index (EI) is then defined as:

$${\text{EI}}={\sum }_{1}^{t}\frac{{\text{PO}}{\text{P}}_{t}\left({E}_{\text{C}}-{E}_{t}\right)}{{{\text{PO}}{\text{P}}_{\text{C}}\left({E}_{\text{C}}\right)}},$$

where ${\text{PO}}{\text{P}}_{t}$ is the population of tract $t$, ${\text{PO}}{\text{P}}_{\text{C}}$ is the population of the county, and the summing takes places across each census tract in the county.

##### Measures of Racial School Segregation

We used three measures of racial school segregation between the non-Hispanic Black and non-Hispanic White school population in each county for each year during the period 1991–2000. First, we used two measures that are parallel to those used to measure residential segregation: the index of dissimilarity and the entropy index. These were calculated as described above, except that the school was the lower unit of geography, and the county was the larger unit. Thus, these measures compared the distribution of the two race/ethnicity groups within each school to the overall distribution of the groups across the entire county.

We also used a third measure of school segregation—the normalized exposure index—which is commonly used to measure school racial segregation [52, 53]. Specifically, we used the Black-White normalized exposure index, which compares the proportion of Black students in the average school attended by Black students in the county to the proportion of Black students in the average school attended by White students in the county. The scale goes from 0 to 1, with 0 indicating no difference in the proportion of Black students in schools attended by White and Black students and 1 indicating complete segregation of Black and White students. The formula for the normalized racism index is

$${\sum }_{s=1}^{n}{P}_{w}\frac{{W}_{s}}{{W}_{c}}-{\sum }_{s=1}^{n}{P}_{w}\frac{{B}_{s}}{{B}_{c}},$$

where ${P}_{w}$ is the percentage of White students in school $s$, ${W}_{s}$ is the number of White students in school $s$, ${W}_{c}$ is the number of White students in the county where school $s$ is located, ${B}_{s}$ is the number of Black students in school $s$, ${B}_{c}$ is the number of Black students in the county, and the summation is over all of the schools in the county.

Data for the school segregation measures were obtained from the Longitudinal School Demographic Dataset (LSDD), a compilation of school demographic statistics compiled by the Sol Price Center for Social Innovation at the University of Southern California [54, 55]. This dataset compiles information from the Common Core of Data published by the National Center for Education Statistics, which has reported annual demographic figures for every public school in the US since the 1986–1987 school year. The LSDD includes enrollment figures—by race/ethnicity—for every public school annually during the period 1991–2020.

In order to ensure that the residential and school segregation indices were reliable, we restricted their estimation to counties that had at least 1000 Black residents in 1991 and at least 500 Black students in 1991. This resulted in a complete set of estimates for the period 1991–2020 for 1051 counties.

##### Choice of Segregation Measures

Our preferred measure for residential segregation was the index of dissimilarity because this is the most used in previous research [12–14, 16]. Our preferred measure for school segregation was the normalized exposure index [52, 53]. However, for the latent trajectory analyses of life expectancy and mortality trends, we used entropy as the residential segregation measure and the index of dissimilarity as the school segregation measure. These measures had the lowest correlation (*r* = 0.68), enabling us to minimize multicollinearity in the trajectory models. There was a strong correlation between the residential segregation measures (correlation coefficient = 0.92 between the index of dissimilarity and the entropy index) and between the school segregation measures (correlation coefficients = 0.94, 0.92, and 0.82 between the index of dissimilarity, the entropy index, and the normalized exposure index).

### Data Analysis

#### Latent Trajectory Analysis

Latent trajectory analysis is a technique for modeling trends in an outcome variable (here, racial segregation and the magnitude of racial disparities in health outcomes) that identifies groups of units (here, counties) that share similar trajectories over time [56–62]. In the context of this paper, identifying trajectory groups for racial segregation and racial disparity outcomes is useful for three purposes: (1) to understand the magnitude of, and trends in racial segregation and in each racial health disparity; (2) to identify which counties exhibit which patterns and therefore which subpopulations are most vulnerable and most in need of intervention; and (3) to examine the relationship between historical levels of and changes in racial segregation and membership in a health disparity trajectory group.

We first conducted a latent multi-trajectory analysis of the trends in residential and school segregation to identify counties with distinct patterns in the levels of and trends in both residential and school segregation. Multi-trajectory analysis is similar to simple trajectory analysis except that it identifies counties with similar patterns in two variables, instead of just one [56–61]. Here, we aimed to identify counties with similar patterns in both racial residential segregation and racial school segregation. After identifying the residential/school segregation trajectory groups, we compared groups in terms of their levels of and changes in each racial health disparity.

We next conducted latent trajectory analyses for each health disparity outcome. After identifying the trajectory groups for each racial health disparity, we used the groups as a dependent variable to examine whether segregation and changes in segregation were predictors of the identified racial disparity trajectories. We explored three questions: (1) Is racial residential and/or school segregation in 1990 predictive of which trajectory group membership during the period 2000–2020? (2) Are residential and school segregation independent predictors of trajectory membership? and (3) After controlling for baseline levels of residential or school segregation, are changes in residential or school segregation from 1990 to 2000 predictive of trajectories in racial health disparities from 2000 to 2020?

We implemented the latent trajectory analysis using the *traj* procedure for STATA, made publicly available by Jones and Nagin [61]. We determined the number of latent groups and the order for the trends (i.e., linear, quadratic, cubic) by visually examining model fit and by inspecting the AIC and BIC statistics for each model.

#### Analytic Strategy

First, we identified patterns in racial residential and school segregation over time (1991–2020) and classified counties into distinct groups that shared similar patterns. We then compared these groups of counties to examine whether they also differed in the level of and trends in racial disparities for each health outcome between 2000 and 2020 and to characterize the relationship between distinct segregation patterns and the level of and trends in these racial health disparities.

Second, for each health outcome, we conducted a latent trajectory analysis to identify trends in racial disparities in that outcome between 2000 and 2020 and classified counties into distinct groups that shared similar patterns. We then examined whether baseline levels of segregation in 1991 and trends in segregation between 1991 and 2000 were significant predictors of which trajectory group each county was assigned. We included four predictor variables in each model: (1) the baseline level of residential segregation in 1991; (2) the baseline level of school segregation in 1991; (3) the change in residential segregation from 1991 to 2000; and (4) the change in school segregation from 1991 to 2000. This allowed us to determine whether changes in segregation between 1991 and 2000 were a significant predictor of trajectory group membership after controlling for baseline levels of segregation in 1991. In addition, to control for the general economic status of each county, we controlled for one time-varying covariate: the percentage of households that were rented each year, obtained from the US decennial censuses and interpolated for intervening years. Finally, because of the large differences in variances across counties due to dissimilarities in population, we weighted the regressions by the square root of the total county population in 2020.

Finally, we ran linear regression models to examine the magnitude of any observed relationship between baseline levels of segregation in 1991 and changes in segregation between 1991 and 2000 and the average level of each racial health disparity during the period 2000–2020. As above, we included four predictor variables in each model: (1) the baseline level of residential segregation in 1991; (2) the baseline level of school segregation in 1991; (3) the change in residential segregation from 1991 to 2000; and (4) the change in school segregation from 1991 to 2000. This allowed us to determine whether changes in segregation between 1991 and 2000 were a significant predictor of the level of each racial health disparity between 2000 and 2020. Because of the large differences in variances across counties due to dissimilarities in population, we weighted the regressions by the square root of the total county population in 2020.

Note that for one outcome—teen birth rate—we skipped the second step (i.e., the latent trajectory analysis) because we had data for only one point in time (2014–2020 combined).

We standardized all independent variables so that the regression coefficient represented the change in the outcome associated with a one standard deviation increase in the independent variable. Analyses were conducted using STATA version 18 (College Station, TX: StataCorp) with the *traj* plug-in [61].

## Results

### Descriptive Results

**Fig. 1:**
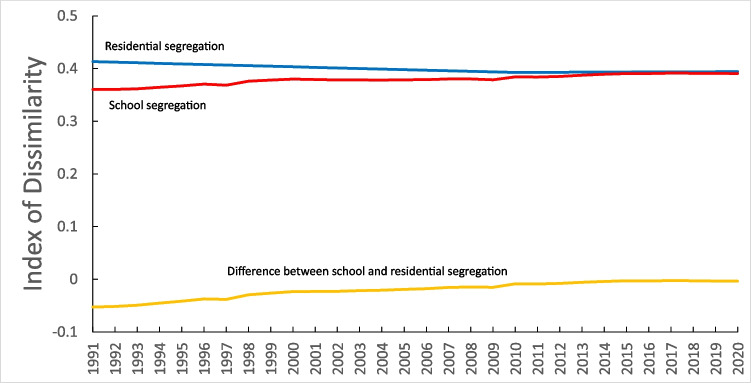
Trends in average racial residential and school segregation—1051 counties, 1991–2020 (non-Hispanic white compared to non-Hispanic Black)

During the period 1991–2020, racial residential segregation remained essentially unchanged among the 1051 counties in our sample, with the residential index of dissimilarity dropping from an average of 0.41 in 1991 to 0.40 in 2020 (Fig. 1). However, racial school segregation increased slightly during this period, with the school index of dissimilarity rising from 0.36 in 1991 to 0.39 in 2020. Thus, the difference between residential segregation and school segregation decreased over the study period, with the average residential index of dissimilarity being 0.05 higher than the average school index of dissimilarity in 1991 but being identical in 2020. Most of the increase in school segregation occurred during the period 1991–2000. Supplemental Fig. 1 is a heat map showing the change in school segregation from 1991 to 2000 for each of the 1051 counties.

### Segregation Multi-trajectory Analysis Results

**Fig. 2:**
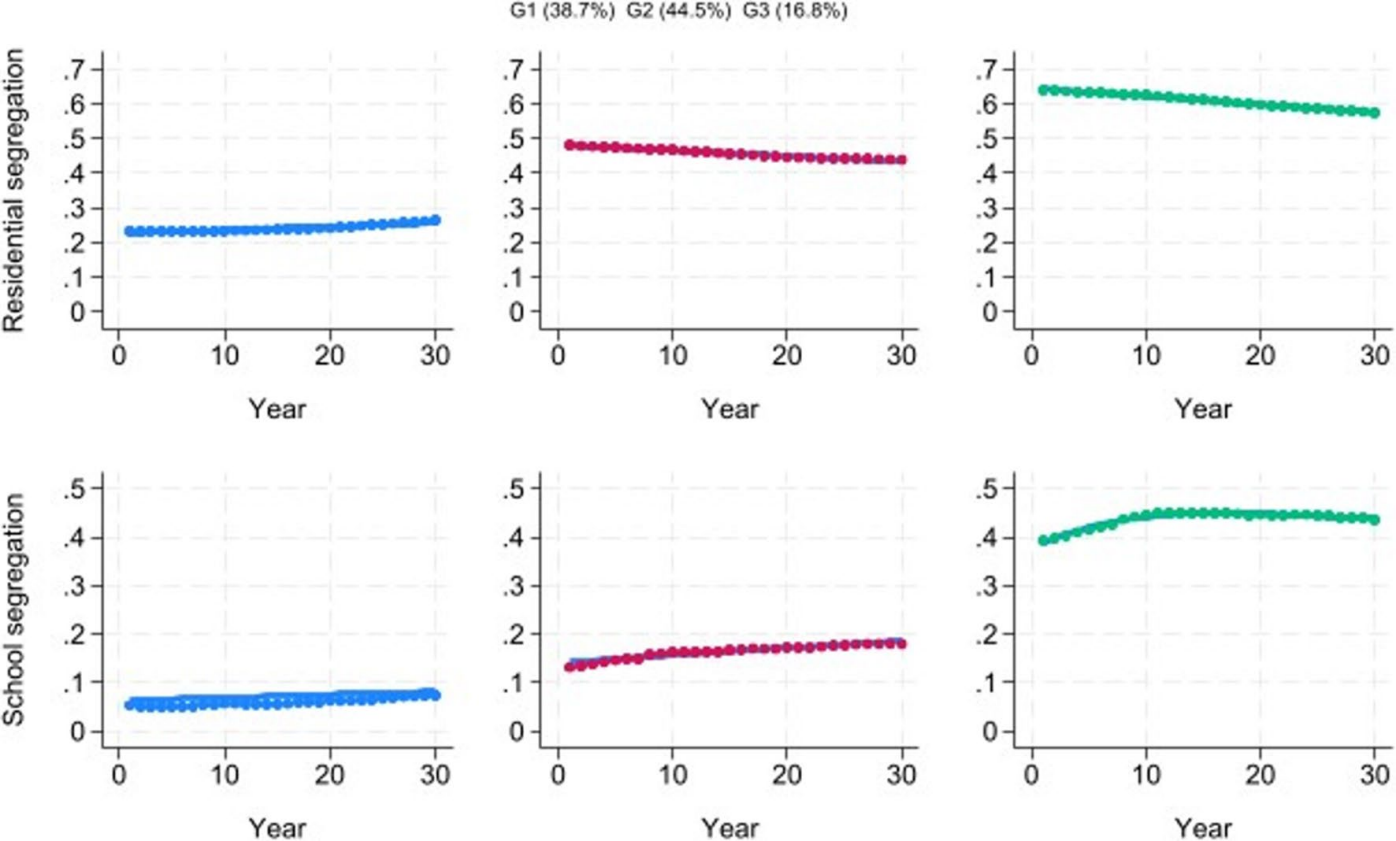
Multi-trajectory analysis of county-level segregation trends, 1991–2020 (year 0 = 1991; year 30 = 2020)

The best-fitting model was a three-group model with linear trends for all groups. Trajectory group 1, which included 38.7% of the counties, was characterized by low school segregation (as measured by the normalized exposure index) that increased slightly from 1991 to 2020 and low residential segregation (as measured by the index of dissimilarity) that also increased slightly over time (Fig. 2). Group 2, which included 44.5% of the counties, was characterized by slightly higher school segregation that increased over time and moderately high residential segregation that declined slightly. Group 3, which included 16.8% of the counties, was characterized by very high school segregation levels that increased from 1991 to 2000 and then remained steady and by very high residential segregation levels that declined slightly during the study period. Therefore, the three groups of counties can be viewed as those with low school and residential segregation and steady trends, those with moderate school and residential segregation and a moderate increase in school segregation over time, and those with very high school and residential segregation and a sharp increase in school segregation from 1991 to 2000.

The average residential index of dissimilarity in 1991 ranged from 0.23 for Group 1, 0.48 for Group 2, and 0.64 for Group 3 (Table 1). The average school normalized exposure index in 1991 ranged from 0.05 for Group 1, 0.13 for Group 2, and 0.39 for Group 3. There was a steady gradation in the change in school segregation between 1991 and 2000, moving from an increase of 0.004 for Group 1, an increase of 0.030 for Group 2, and an increase of 0.053 for Group 3.

**Table 1:** Racial segregation and racial health disparity profile for each residential/school segregation multi-trajectory group and geodemographic characteristics of each group

	Group 1	Group 2	Group 3
Segregation pattern			
Percentage of counties in group	38.7%	44.5%	16.8%
Residential segregation, baseline 1991	Low	Moderate	High
Residential segregation, trend 1991–2020	Slight increase	Slight decrease	Slight decrease
School segregation, baseline 1991	Low	Moderate	High
School segregation, trend 1991–2020	Slight increase	Slight increase	Large increase, 1991–2000, then steady
Average residential index of dissimilarity, 1991	0.23	0.48	0.64
Average change in index of dissimilarity, 1991–2000	–0.001	–0.013	–0.020
Average change in index of dissimilarity, 1991–2020	+ 0.029	–0.041	–0.070
Average school normalized exposure index, 1991	0.05	0.13	0.39
Average change in school normalized exposure index, 1991–2000	+ 0.004	+ 0.030	+ 0.053
Average change in school normalized exposure index, 1991–2020	+ 0.021	+ 0.047	+ 0.043
Racial health disparities			
Average Black-White life expectancy differential, 2000–2019	2.8	3.3	4.4
Average life expectancy differential, 2019	2.3	3.0	4.1
Average change in life expectancy differential, 2000–2019	–1.8	–1.5	–1.4
Average Black-White age-adjusted early mortality ratio, 2000–2020	1.38	1.49	1.71
Average early mortality ratio, 2020	1.35	1.52	1.89
Average change in early mortality ratio, 2000–2020	–0.29	–0.21	+ 0.01
Average Black-White infant mortality rate ratio, 2001–2018	2.11	2.39	2.67
Average infant mortality rate ratio, 2018	1.95	2.56	3.15
Average change in infant mortality rate ratio, 2001–2018	+ 0.02	+ 0.10	+ 0.47
Average Black-White firearm homicide rate ratio, 2002–2017	N/A	7.19	12.39
Average firearm homicide rate ratio, 2017	N/A	7.85	13.67
Average change in firearm homicide rate ratio, 2002–2017	N/A	+ 0.77	+ 2.00
Average Black-White total homicide rate ratio, 2002–2017	3.97	5.51	8.77
Average total homicide rate ratio, 2017	3.91	6.10	9.91
Average change in total homicide rate ratio, 2002–2017	+ 0.73	+ 0.82	+ 2.52
Average Black-White teen birth rate ratio, 2014–2020	1.48	1.94	3.76
Geodemographic characteristics			
Median total population, 2020	27,000	106,000	351,000
Median population density, 2020	56	176	603
Average percentage of Black population, 2020	26.6%	16.5%	22.4%
Average percentage living in rental housing, 2020	29.6%	32.7%	38.4%
Average percentage without college degree, 2020	79.1%	74.4%	68.7%
Average poverty rate, 2020	17.4%	15.6%	15.3%
Average unemployment rate, 2020	6.3%	5.9%	6.1%
Percentage of counties in Northeast Census region	0.7%	9.0%	25.6%
Percentage of counties in Midwest Census region	2.5%	14.3%	26.1%
Percentage of counties in South Census region	91.9%	68.9%	43.2%
Percentage of counties in West Census region	4.9%	7.9%	5.1%

*N/A*, there were no counties in this trajectory group with health outcome data available for this variable

The gradation in residential and school segregation moving from Group 1 to Group 2 to Group 3 was matched by a similar pattern of differences between trajectory groups in the average level of the racial health disparity for every one of the health outcomes (Table 1). For example, the average Black-White life expectancy differential increased from 2.8 for Group 1 to 3.3 for Group 2 to 4.4 for Group 3. The average Black-White early mortality ratio increased from 1.38 for Group 1 to 1.49 for Group 2 to 1.71 for Group 3. The average Black-White infant mortality rate ratio increased from 2.11 for Group 1 to 2.39 for Group 2 to 2.67 for Group 3. A similar pattern was observed for racial disparities in total homicide (3.97 to 5.51 to 8.77) and teen birth rate (1.48 to 1.94 to 3.76).

Finally, for each racial health disparity, there was a gradation in the degree of change in the level of disparity from 2000 to 2020 moving from Group 1 to Group 2 to Group 3, with higher groups experiencing either less of a decline in the disparity or more of an increase in the disparity (Table 1). For example, the change in the life expectancy differential was a decline of 1.8 years for Group 1, a decline of 1.5 years for Group 2, and a decline of 1.4 years for Group 3. The change in the early mortality rate ratio was a decline of 0.29 for Group 1, a decline of 0.29 for Group 2, and an increase of 0.01 for Group 3. The change in the infant mortality rate ratio was an increase of 0.02 for Group 1, an increase of 0.10 for Group 2, and an increase of 0.47 for Group 3. For the total homicide rate ratio, the increases were 3.97, 5.51, and 8.77. For the teen birth rate ratio, the increases were 1.48, 1.94, and 3.76.

Sociodemographically, counties in Group 3 were largely urban counties with high population density and greater population size (Table 1). They tended to have slightly higher levels of educational attainment. Geographically, Group 3 counties were much more likely than other groups to be in the Northeast and Midwest. Group 1 counties were largely in the South, while Group 2 counties were predominantly in the South and Midwest. Supplemental Fig. 2 is a heat map showing which counties were in each multi-trajectory group.

The 50 counties with the highest levels of school segregation at baseline in 1991 are listed in Table 2. The average normalized exposure index for these 50 counties was 0.59, compared to 0.12 for all other counties combined. Most of these counties had large Black-White life expectancy differentials. The highest differentials were 15.5 years in Washington, DC, 8.2 years in Cook County, Illinois (Chicago), 7.3 years in Fulton County, Georgia (Atlanta), 7.1 years in Mahoning County, Ohio (Youngstown), and 6.8 years in Essex County, New Jersey (Newark) and Alameda County, California (Oakland). The average life expectancy differential for the 50 counties with the highest baseline levels of school segregation was 4.6 years, compared to 2.8 years for all other counties combined. Similar patterns were observed for each of the other racial health disparities. Perhaps, the most striking finding was the extremely high racial disparity in firearm homicide rates, with rate ratios of 42.3 in Cook County, Illinois, 20.2 in Fulton County, Georgia, 26.3 in Monroe County, New York (Rochester), and 23.5 in Baltimore. Also striking was the finding that the 50 most segregated counties had an average Black-White teen birth rate ratio of 4.81 compared to 1.95 for all other counties combined.

**Table 2:** Summary of school segregation levels and racial health disparity levels and trends for top 50 counties in terms of their Black-White school segregation levels in 1991

County (state)	School segregation, 1991 (normalized exposure index)* N = 1051	Black-White life expectancy differential, 2019 (Black minus White) *N* = 1051	Black-White early mortality rate ratio, 2020 *N* = 538	Black-White infant mortality rate ratio, 2018 *N* = 199	Black-White firearm homicide rate ratio, 2017 *N* = 104	Black-White total homicide rate ratio, 2017 *N* = 183	Black-White teen birth rate ratio, 2014–2020 (combined) *N* = 1019
Top 50 counties in terms of Black-White school segregation in 1991, as measured by normalized exposure index							
Lake County (Indiana)	0.86	− 4.9	1.74	2.81	13.78	10.08	2.67
Wayne County (Michigan)	0.82	− 5.7	2.25	2.85	15.78	12.70	3.13
Essex County (New Jersey)	0.79	− 6.8	2.76	2.96			9.02
Passaic County (New Jersey)	0.77	− 5.9	2.53	1.50			9.85
Cook County (Illinois)	0.74	− 8.2	3.01	3.77	42.30	27.59	8.41
Saginaw County (Michigan)	0.71	− 6.2	2.26			12.79	3.88
Saint Clair County (Illinois)	0.69	− 6.1	2.11	2.86		20.00	3.44
Mahoning County (Ohio)	0.69	− 7.1	1.84	4.85		14.91	4.25
Hudson County (New Jersey)	0.69	− 4.1	1.96	2.57			5.24
Jefferson County (Alabama)	0.68	− 3.4		2.37	8.84	7.79	2.91
Hancock County (Georgia)	0.68	− 2.5	1.59				
Genesee County (Michigan)	0.67	− 5.1	2.07	2.93	17.96	12.26	2.62
Fulton County (Georgia)	0.66	− 7.3	3.06	4.00	20.18	15.41	12.02
Nassau County (New York)	0.63	− 1.9	1.64	2.99		11.50	7.13
Hamilton County (Tennessee)	0.63	− 4.8	1.63	2.62	10.00	11.00	2.27
Mercer County (New Jersey)	0.61	− 6.5	2.30				9.91
Muskegon County (Michigan)	0.60	− 6.1	2.23				2.79
Dauphin County (Pennsylvania)	0.59	− 5.0	2.00	2.43			2.69
Camden County (New Jersey)	0.58	− 4.5	1.90	4.50		14.41	3.83
Alameda County (California)	0.58	− 6.8	2.74	2.64	17.80	14.14	4.60
Berrien County (Michigan)	0.58	− 6.4	1.96	2.53			3.45
Ouachita Parish (Louisiana)	0.57	− 4.5	1.88	2.61		4.79	2.22
Madison County (Mississippi)	0.57	− 3.9	2.26				4.28
Hartford County (Connecticut)	0.57	− 1.8	1.61	3.19		10.62	4.44
Kings County (New York)	0.57	− 3.0	2.03	1.66	15.17	8.64	1.67
Shelby County (Tennessee)	0.57	− 5.2	1.83	2.45	9.60	8.02	4.30
Cuyahoga County (Ohio)	0.56	− 5.0	1.90	4.40	15.52	12.60	4.56
Delaware County (Pennsylvania)	0.56	− 3.6	1.78	3.25		16.75	5.10
Halifax County (North Carolina)	0.56	− 2.5	1.56				1.77
Atlantic County (New Jersey)	0.55	− 5.2	1.54	3.99		10.33	5.39
Los Angeles County (California)	0.54	− 5.5	2.23	2.88	12.60	9.54	6.26
Queens County (New York)	0.54	− 1.4	1.42	1.73		5.85	2.35
District of Columbia	0.53	− 15.5	7.07	7.98			36.82
Coahoma County (Mississippi)	0.53	− 4.8					2.18
Monroe County (New York)	0.52	− 6.1	2.79	4.11	26.29	18.50	5.78
Oakland County (Michigan)	0.52	− 4.0	2.08	2.46	16.75	11.00	3.78
New Haven County (Connecticut)	0.51	− 2.8	1.71	2.67		13.75	4.47
Union County (New Jersey)	0.51	− 3.6	2.05				8.73
Harris County (Texas)	0.51	− 4.7	1.70	2.66	6.63	5.60	2.38
Dallas County (Texas)	0.51	− 4.2	1.60	2.47	7.38	5.81	2.43
Philadelphia County (Pennsylvania)	0.51	− 3.8	1.60	3.44	14.79	10.31	3.78
Baltimore City (Maryland)	0.51	− 5.3	1.63	2.92	23.48	12.09	3.04
Osage County (Oklahoma)	0.51	+ 5.5					0.30
Bronx County (New York)	0.50	− 0.8	1.13	1.53		5.00	1.66
Jackson County (Missouri)	0.50	− 6.1	1.87	2.44	10.98	9.83	2.19
Montgomery County (Ohio)	0.49	− 4.2	1.60	2.40	13.56	10.08	2.20
Jefferson County (Texas)	0.49	− 3.8	1.43	2.18		3.45	1.93
Kankakee County (Illinois)	0.49	− 5.3	1.84				4.80
Lincoln County (Arkansas)	0.49	+ 1.0					1.12
Calcasieu Parish (Louisiana)	0.48	− 4.0	1.47	2.54		4.14	1.48
***All 50 counties (average)***	***0.59***	*** − 4.6***	***2.07***	***3.00***	***15.97***	***11.21***	***4.81***
**All other counties (average)**	**0.12**	** − 2.8**	**1.55**	**2.79**	**9.87**	**7.51**	**1.95**

^*^The normalized exposure index scale goes from 0 to 1, with 0 indicating no difference in the proportion of Black students in each school compared to the proportion of Black students in the district and 1 indicating complete segregation of Black and White students in separate schools

#### Life Expectancy Disparity Trends

**Fig. 3:**
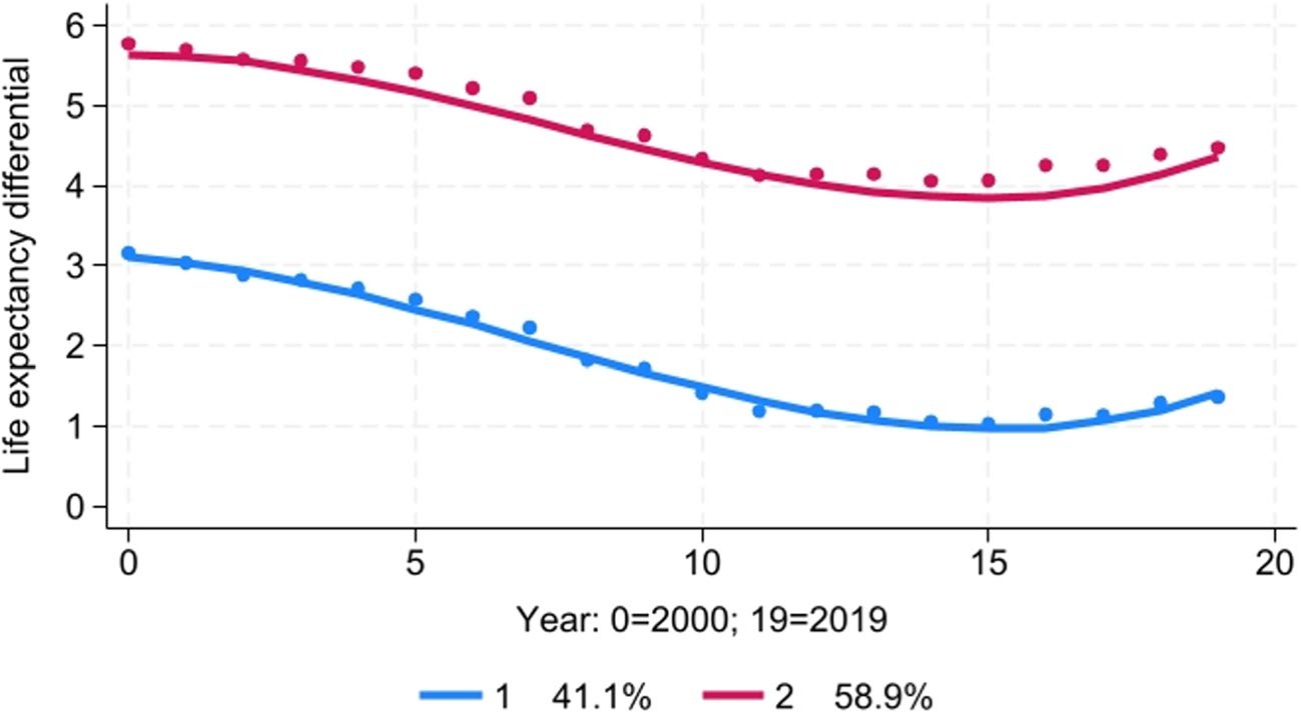
Trajectory analysis of Black-White life expectancy differential, 2000–2019

The best-fitting model was a two-group model with a cubic trend pattern for both groups (Fig. 3). The trends in the Black-White life expectancy differential were roughly parallel; however, Group 2 had a much higher average disparity than Group 1, roughly 3 years higher during the study period. In both groups, the racial disparity in life expectancy declined steadily from 2000 to 2014 but increased from 2015 to 2019. Approximately 41% of the counties were classified in Group 1, while 59% were classified in Group 2.

Higher levels of baseline residential segregation in 1991 were a significant predictor of the likelihood a county was classified in Group 2 compared to Group 1 (Table 3). After controlling for baseline residential segregation, greater increases in school segregation between 1991 and 2000 were significantly associated with the likelihood of a county being classified in Group 2.

**Table 3:** Results of latent trajectory analysis: effect of racial residential and school segregation (baseline 1991 and change 1991–2000) on the likelihood of trajectory group membership for trends in each racial health disparity, 2000–2020

Group	Parameter	Odds ratio	95% CI	*p*-value
**Effect of racial segregation on the likelihood of trajectory group membership for trends in racial disparity in life expectancy, 2000–2019**				
Group 1	Reference group	1.00	–-	–-
Group 2	**Residential segregation, 1991**	**1.98**	**1.35 − 2.90**	** < 0.001**
	School segregation, 1991	1.14	0.90 − 1.45	0.28
	Change in residential segregation, 1991–2000	0.89	0.72 − 1.10	0.27
	**Change in school segregation, 1991–2000**	**1.26**	**1.03 − 1.54**	**0.025**
**Effect of racial segregation on the likelihood of trajectory group membership for trends in racial disparity in early mortality, 2000–2020**				
Group 1	Reference group	1.00	–-	–-
Group 2	Residential segregation, 1991	1.03	0.66 − 1.62	0.88
	**School segregation, 1991**	**2.40**	**1.50 − 3.83**	** < 0.001**
	Change in residential segregation, 1991–2000	0.81	0.63 − 1.05	0.11
	**Change in school segregation, 1991**–**2000**	**1.36**	**1.01 − 1.82**	**0.043**
**effect of racial segregation on the likelihood of trajectory group membership for trends in racial disparity in infant mortality, 2001–2018**				
Group 1	Reference group	1.00	–-	–-
Group 2	Residential segregation, 1991	1.70	0.98–2.93	0.058
	School segregation, 1991	1.64	0.97–2.78	0.065
	Change in residential segregation, 1991–2000	0.74	0.51–1.08	0.12
	**Change in school segregation, 1991–2000**	**2.02**	**1.18–3.48**	**0.011**
**Effect of racial segregation on the likelihood of trajectory group membership for trends in racial disparity in firearm homicide, 2002–2017**				
Group 1	Reference group	1.00	–-	–-
Group 2	Residential segregation, 1991	1.44	0.55–3.71	0.46
	**School segregation, 1991**	**5.47**	**1.77**–**17.0**	**0.003**
	Change in residential segregation, 1991–2000	0.73	0.42–1.27	0.27
	**Change in school segregation, 1991**–**2000**	**2.26**	**1.08**–**4.74**	**0.030**
**effect of racial segregation on the likelihood of trajectory group membership for trends in racial disparity in total homicide, 2002–2017**				
Group 1	Reference group	1.00	–-	–-
Group 2	Residential segregation, 1991	0.95	0.42–2.19	0.91
	**School segregation, 1991**	**10.28**	**3.75**–**28.2**	** < 0.001**
	Change in residential segregation, 1991–2000	0.60	0.35–1.02	0.061
	**Change in school segregation, 1991**–**2000**	**3.32**	**1.72**–**6.42**	** < 0.001**

Coefficients that are statistically significant at *p* < 0.05 are shown in bold type

All predictor variables are standardized. Therefore, odds ratios indicate the ratio of the likelihood of being in the trajectory group compared to the reference group associated with each one standard deviation increase in the predictor variable

The measure used here for residential segregation was the index of dissimilarity

The measure used here for school segregation was the normalized exposure index

In a linear regression model, both residential and school segregation in 1991 were found to be significant independent predictors of the average Black-White life expectancy differential during the period 2000–2019 (Table 4). After controlling for both residential and school segregation levels at baseline, greater increases in school segregation between 1991 and 2000 were significantly associated with a higher life expectancy differential between 2000 and 2019.

**Table 4:** Results of linear regression models to examine the relationship between baseline levels of segregation in 1991 and changes in segregation between 1991 and 2000 and the average level of each racial health disparity during the period 2000–2020

Disparity	Variable (all are standardized)	Regression coefficient (95% CI)	*p*-value
Life expectancy (*N* = 1051 counties)			
Average Black-White life expectancy differential, 2000–2019	**Residential segregation, 1991**	** + 0.41 (+ 0.22 to + 0.60)**	** < 0.001**
	**School segregation, 1991**	** + 0.44 (+ 0.30 to + 0.59)**	** < 0.001**
	Change in residential segregation, 1991–2000	− 0.01 (− 0.13 to + 0.11)	0.89
	**Change in school segregation, 1991**–**2000**	** + 0.15 (+ 0.01 to + 0.30)**	**0.041**
Early mortality (*N* = 538 counties)			
Average Black-White early mortality rate ratio, 2000–2020	**Residential segregation, 1991**	** + 0.08 (+ 0.02 to + 0.14)**	**0.013**
	**School segregation, 1991**	** + 0.10 (+ 0.06 to + 0.14)**	** < 0.001**
	Change in residential segregation, 1991–2000	+ 0.00 (− 0.04 to + 0.04)	0.92
	**Change in school segregation, 1991–2000**	** + 0.04 (+ 0.00 to + 0.08)**	**0.033**
Infant mortality (*N* = 199 counties)			
Average Black-White infant mortality rate ratio, 2001–2018	**Residential segregation, 1991**	** + 0.18 (+ 0.07 to + 0.30)**	**0.002**
	School segregation, 1991	+ 0.00 (− 0.10 to + 0.11)	0.95
	Change in residential segregation, 1991–2000	− 0.00 (− 0.05 to + 0.05)	0.99
	**Change in school segregation, 1991–2000**	** + 0.09 (+ 0.01 to + 0.16)**	**0.023**
Firearm homicide (*N* = 104 counties)			
Average Black-White firearm homicide rate ratio, 2002–2017	**Residential segregation, 1991**	** + 2.55 (+ 1.19 to + 3.91)**	** < 0.001**
	**School segregation, 1991**	** + 1.71 (+ 0.04 to + 3.38)**	**0.044**
	Change in residential segregation, 1991–2000	− 0.65 (− 1.40 to + 0.10)	0.087
	**Change in school segregation, 1991–2000**	** + 1.29 (+ 0.25 to + 2.33)**	**0.015**
Total homicide (*N* = 183 counties)			
Average Black-White total homicide rate, 2002–2017	**Residential segregation, 1991**	** + 1.41 (+ 0.78 to + 2.05)**	** < 0.001**
	**School segregation, 1991**	** + 1.07 (+ 0.31 to + 1.83)**	**0.006**
	**Change in residential segregation, 1991–2000**	** − 0.44 (**− **0.79 to − 0.09)**	**0.013**
	**Change in school segregation, 1991–2000**	** + 0.87 (+ 0.36 to 1.39)**	**0.001**
Teen birth rate			
Average Black-White teen birth rate ratio, 2014–2020 (combined)	**Residential segregation, 1991**	** + 0.44 (+ 0.11 to + 0.77)**	**0.010**
	**School segregation, 1991**	** + 0.68 (+ 0.44 to + 0.91)**	** < 0.001**
	Change in residential segregation, 1991–2000	+ 0.01 (− 0.30 to + 0.50)	0.97
	**Change in school segregation, 1991–2000**	** + 0.26 (+ 0.01 to + 0.50)**	**0.040**

Coefficients that are statistically significant at p < 0.05 are shown in bold type

Regression coefficients indicate the change in the outcome variable for each one standard deviation increase in the predictor variable

The measure used here for residential segregation was the index of dissimilarity

The measure used here for school segregation was the normalized exposure index

#### Early Mortality Disparity Trends

**Fig. 4:**
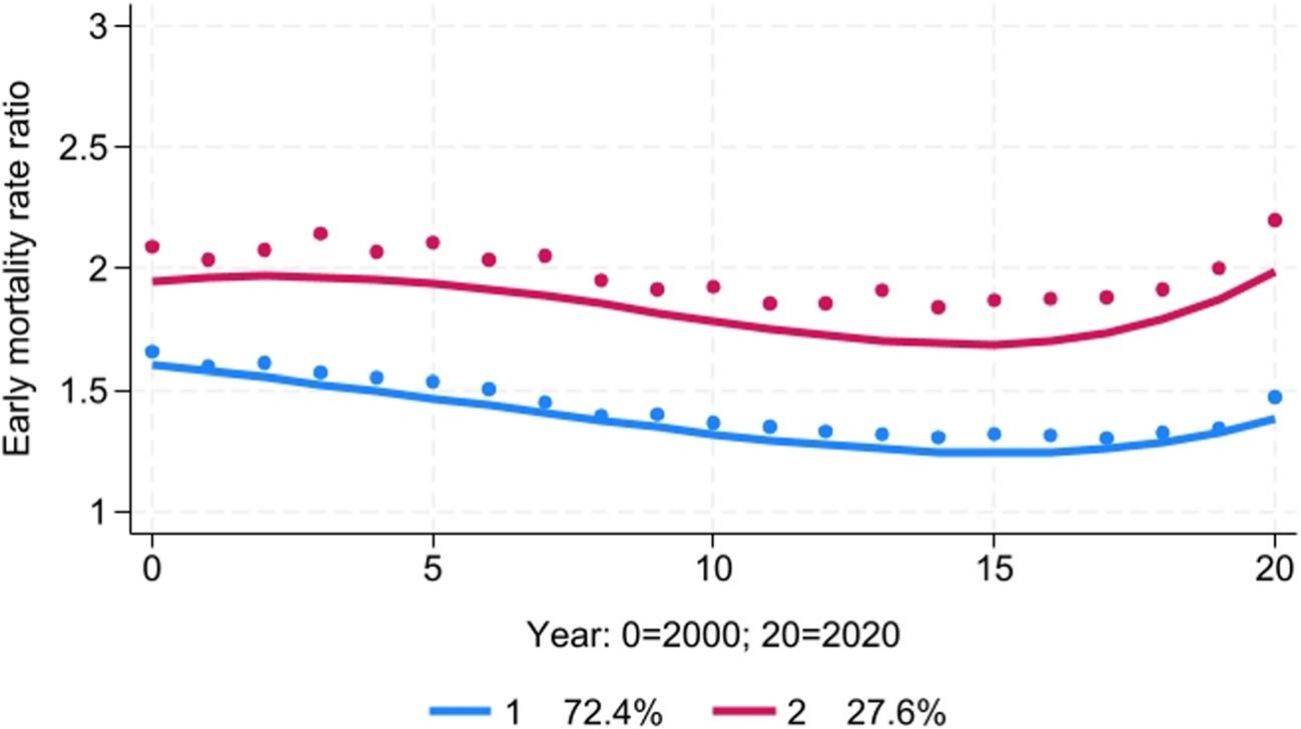
Trajectory analysis of Black-White early mortality rate ratio, 2000–2020

The best-fitting model was a two-group model with a quartic trend pattern for both groups (Fig. 4). The trends in the Black-White early mortality rate ratio were roughly parallel, but the racial disparity was substantially higher among counties in Group 2. The average early mortality rate ratio hovered around 1.5 for Group 1 and around 2.0 for Group 2. The racial disparity in early mortality declined among both groups from 2000 to 2014 but increased from 2015 to 2020, such that by 2020, the average early mortality rate ratio was 1.5 for Group 1 and 2.2 for Group 2, the widest differential for any year in the study period. Approximately 72% of the counties were classified in Group 1, while 28% were classified in Group 2.

Higher levels of baseline school segregation in 1991 were a significant predictor of the likelihood a county was classified in Group 2 compared to Group 1 (Table 3). After controlling for baseline school segregation, greater increases in school segregation between 1991 and 2000 were significantly associated with the likelihood of a county being classified in Group 2.

In a linear regression model, both residential and school segregation in 1991 were found to be significant independent predictors of the average Black-White early mortality rate ratio during the period 2000–2020 (Table 4). After controlling for both residential and school segregation levels at baseline, greater increases in school segregation between 1991 and 2000 were significantly associated with a higher early mortality rate ratio between 2000 and 2020.

#### ***Infant Mortality Disparity Trends***

**Fig. 5:**
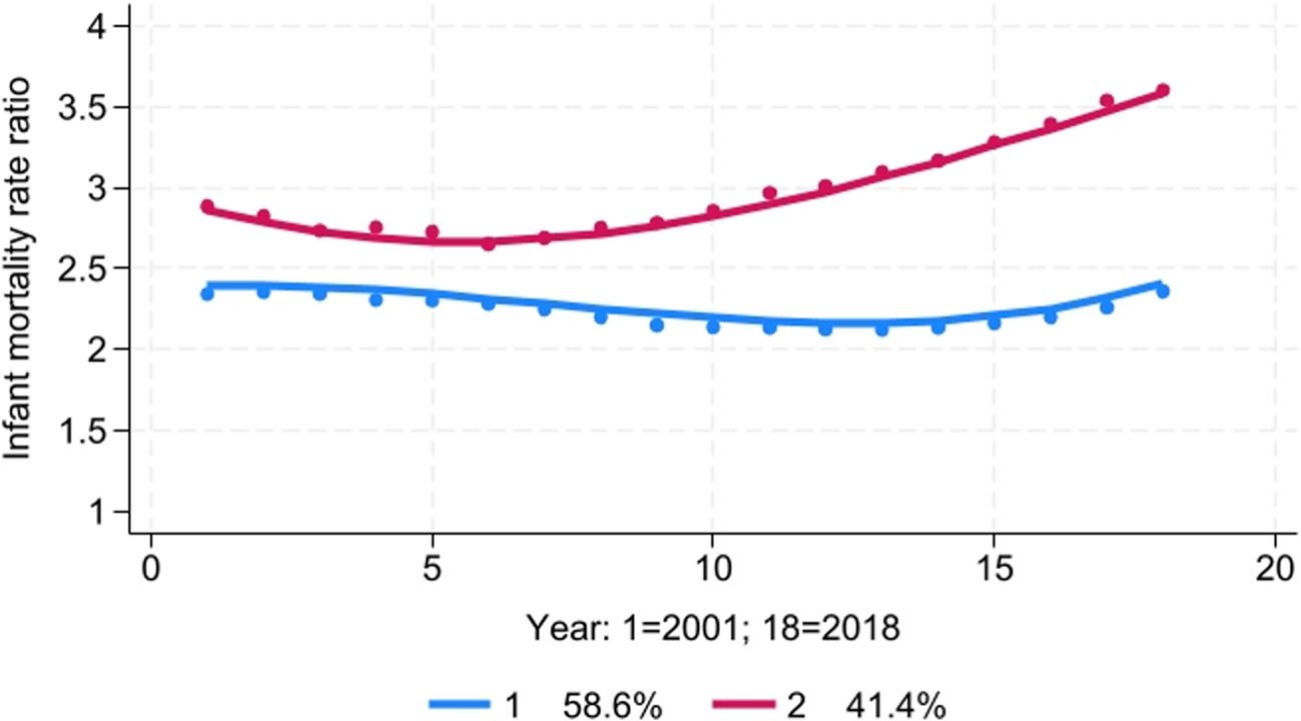
Trajectory analysis of Black-White infant mortality rate ratio, 2001–2018

The best-fitting model was a two-group model with a cubic trend for each group (Fig. 5). Group 1 was characterized by moderately high racial disparities in infant mortality, with averages ranging from 2.1 to 2.4. There was a slight decline in the magnitude of this disparity from 2001 to 2013 and a slight increase from 2014 to 2018. Group 2 was characterized by extremely high racial disparities in infant mortality, with averages ranging from 2.6 to 3.6. There was a small decline in the Black-White infant mortality rate ratio for Group 2 from 2001 to 2006 but a stark and steady increase from an average of 2.6 in 2006 to 3.6 in 2018.

Higher levels of baseline residential and school segregation in 1991 were positive, but not statistically significant predictors of the likelihood a county was classified in Group 2 compared to Group 1 (Table 3). However, after controlling for baseline residential and school segregation, greater increases in school segregation between 1991 and 2000 were significantly associated with the likelihood of a county being classified in Group 2.

In a linear regression model, residential segregation in 1991 was found to be a significant predictor of the average Black-White infant mortality rate ratio during the period 2001–2018 (Table 4). After controlling for both residential and school segregation levels at baseline, greater increases in school segregation between 1991 and 2000 were significantly associated with a higher infant mortality rate ratio between 2001 and 2018.

#### Firearm Homicide Disparity Trends

**Fig. 6:**
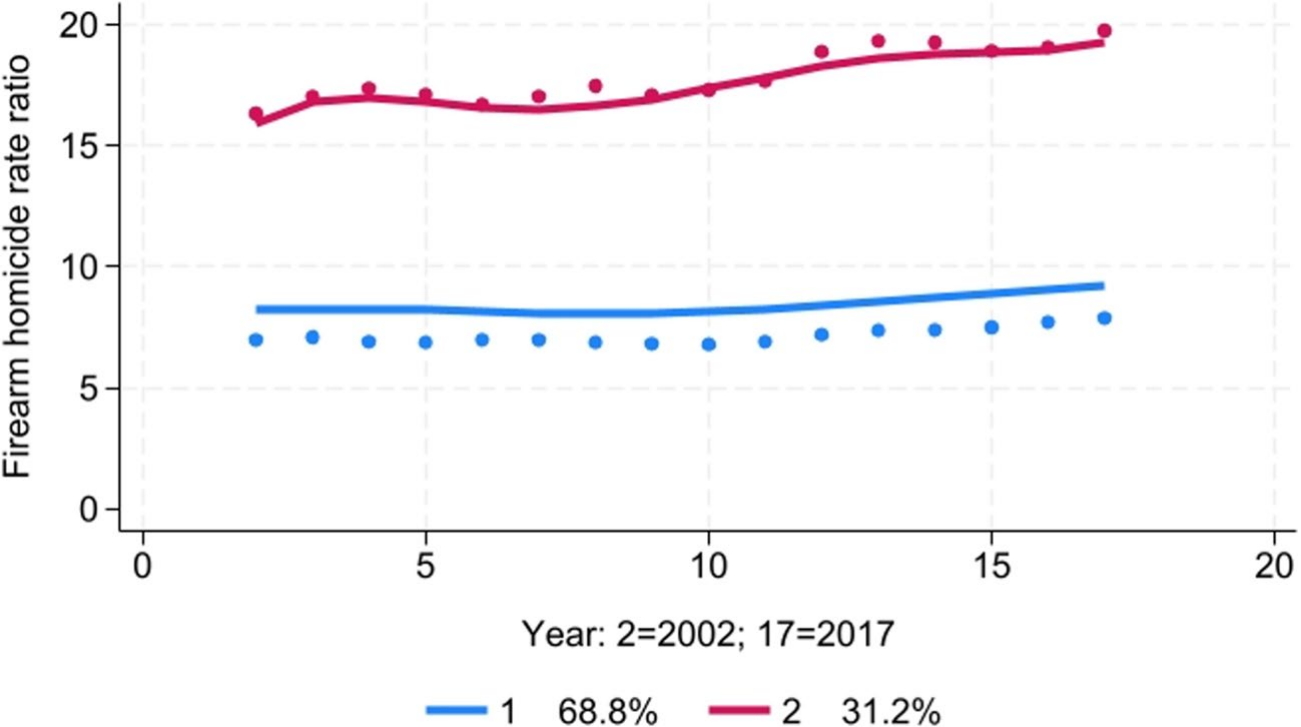
Trajectory analysis of Black-White firearm homicide rate ratio, 2002–2017

The best-fitting model was a two-group model with a quintic trend for each group (Fig. 6). Group 1 (69% of the 104 counties) was characterized by average Black-White total homicide rate ratios of approximately seven, relatively steady over time but with a slight uptick between 2013 and 2017. Group 2 (31%) was characterized by very high disparities (rate ratios of greater than 15) and by increasing rate ratios from 2010 to 2017.

Higher levels of baseline school segregation in 1991 were a significant predictor of the likelihood a county was classified in Group 2 compared to Group 1 (Table 3). After controlling for baseline residential and school segregation, greater increases in school segregation between 1991 and 2000 were significantly associated with the likelihood of a county being classified in Group 2.

In a linear regression model, both residential and school segregation in 1991 were found to be significant independent predictors of the average Black-White firearm homicide rate ratio during the period 2002–2017 (Table 4). After controlling for both residential and school segregation levels at baseline, greater increases in school segregation between 1991 and 2000 were significantly associated with a higher firearm homicide rate ratio between 2002 and 2017.

#### Total Homicide Disparity Trends

**Fig. 7:**
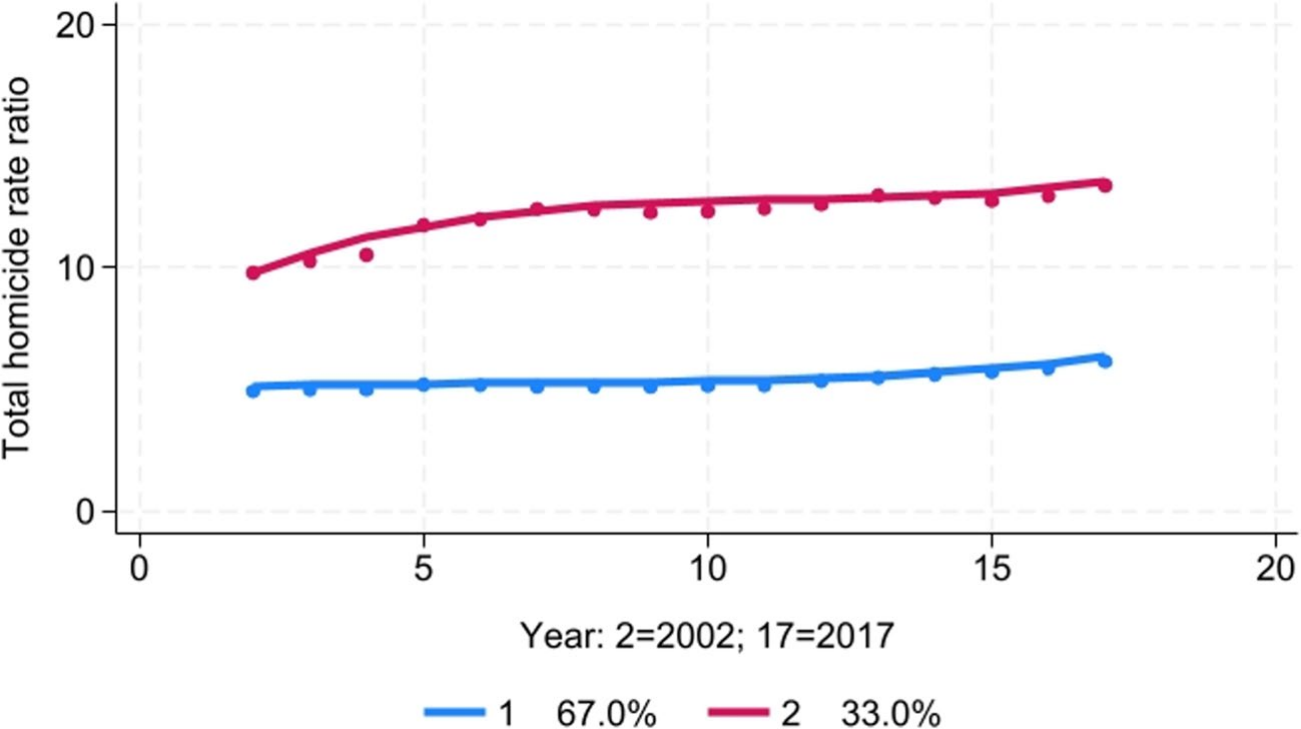
Trajectory analysis of Black-White total homicide rate ratio, 2002–2017

The best-fitting model was a two-group model with a cubic trend for each group (Fig. 7). Group 1 (67% of the 183 counties) was characterized by average Black-White total homicide rate ratios of approximately five, relatively steady over time but with a slight uptick between 2013 and 2017. Group 2 (33%) was characterized by higher disparities (rate ratios of greater than 10) and by increasing rate ratios over the study period.

Higher levels of baseline school segregation in 1991 were a significant predictor of the likelihood a county was classified in Group 2 compared to Group 1 (Table 3). After controlling for baseline residential and school segregation, greater increases in school segregation between 1991 and 2000 were significantly associated with the likelihood of a county being classified in Group 2.

In a linear regression model, both residential and school segregation in 1991 were found to be significant independent predictors of the average Black-White total homicide rate ratio during the period 2002–2017 (Table 4). After controlling for both residential and school segregation levels at baseline, greater increases in school segregation between 1991 and 2000 were significantly associated with a higher total homicide rate ratio between 2002 and 2017, while greater declines in residential segregation were associated with a higher total homicide rate ratio during the study period.

#### Teen Birth Rate Ratio

In a linear regression model, both residential and school segregation in 1991 were found to be significant independent predictors of the average Black-White teen birth rate ratio during the period 2014–2020 (Table 4). After controlling for both residential and school segregation levels at baseline, greater increases in school segregation between 1991 and 2000 were significantly associated with a higher teen birth rate ratio between 2014 and 2020.

#### Case Studies: Relating Policy to Segregation Data

To better understand the impact of county-level policies on racial segregation in schools, we closely examined residential and school segregation trends in two counties whose school segregation policies have been articulated in great detail: Mecklenburg and Wake counties in North Carolina [63]. Both counties are notable for having committed to desegregating their schools in the 1970s [63]. However, in the 1990s, both counties enacted policies to resegregate schools, ending busing and formally ending integration after court rulings gave them legal clearance to resegregate (63). Our data are consistent with this history; in both counties, school segregation was substantially lower than residential segregation in 1991, but by 2020, this gap had disappeared. While residential segregation in Mecklenburg County remained relatively steady at around 0.6 (index of dissimilarity) during the entire period 1991–2020, school integration increased from 0.25 to 0.61, completely eliminating the gap between school and residential segregation levels. Similarly, in Wake County, residential segregation remained stable at around 0.45 between 1991 and 2020, but school integration increased from 0.20 to 0.40, almost completely eliminating the gap between school and residential segregation levels.

## Discussion

To the best of our knowledge, this is the first paper to simultaneously evaluate the impact of both residential and school racial segregation on racial health disparities over a 30-year period and the first to examine the relationship between changes in residential and school segregation and subsequent trends in these racial health disparities. Several major findings emerged from this study.

First, there has been very little overall progress in reducing residential racial segregation in the United States. However, there has been a substantial increase in school racial segregation, resulting in a complete elimination of the gap between school and residential segregation that existed in 1991. We identified one group of counties, representing 17% of our sample, that are characterized by extremely high levels of both residential and school segregation and in which school segregation increased between 1991 and 2000 with no subsequent decline through the year 2020.

Second, counties with worse trajectory patterns (i.e., higher levels of residential and school segregation and greater increases in school segregation over time) have experienced higher levels of Black-White racial disparities in several health outcomes across the life course as well as less progress in reducing these disparities. This relationship holds for health outcomes in adolescence (teen pregnancy rates), young adulthood (infant mortality and homicide), adulthood (early mortality), and throughout the lifespan (life expectancy).

Third, we found evidence that for some racial health disparities (life expectancy, early mortality, firearm homicide, total homicide, and teen pregnancy), residential and school segregation are independent predictors of worse trajectories in these disparities over a subsequent time period. Even after controlling for levels of residential segregation, higher levels of school segregation were significantly predictive of higher levels of and less progress in reducing racial health disparities.

Fourth, we found consistent evidence that for each of the racial health disparities examined, increases in school segregation between 1991 and 2000 were associated with higher levels of and less progress in reducing racial health disparities during the subsequent two decades (2000–2020). This association was present even after controlling for baseline levels of both residential and school segregation in 1991. The change in school segregation between 1991 and 2000 was therefore a robust predictor of trajectories in racial health disparities between 2000 and 2020.

Finally, we identified a disturbing pattern of increases in already high levels of racial health disparities from approximately 2010 forward or 2015 forward among counties in higher trajectory groups (i.e., groups with higher levels of these racial health disparities). The latent trajectory analyses revealed that the higher trajectory groups experienced increases in racial disparities in life expectancy from 2015 to 2019, in early mortality from 2015 to 2020, in infant mortality from 2009 to 2018, in firearm homicide from 2009 to 2017, and in total homicide from 2010 to 2017.

It should not be inferred that changes in residential segregation do not affect racial health disparities. It is possible that the slight changes in residential segregation over the study period provide little variation between counties, while the primary change that provides variation in the data is the resegregation of many US schools that occurred in the 1990s. In fact, preliminary evidence suggests that changes in residential segregation are associated with distinct trajectories of Black-White disparities in early mortality at the metropolitan level [64].

There are various mechanisms by which racial school segregation can confer differential disadvantage on Black schoolchildren and advantage on White schoolchildren that may accumulate over the life course to result in observable racial health disparities in adolescence, young adulthood, or later adulthood. Racially segregated schools tend to receive less public funding, resulting in overcrowded classrooms, fewer educational resources, less trained and experienced teachers, and fewer educational opportunities [37, 65–68]. These schools may also affect students’ self-esteem by influencing their view of where they are situated in the social hierarchy [37, 69]. Racially segregated schools may also result in the exposure of Black youth to environmental stressors, such as higher rates of punishment, disorder, and violence [37, 70]. These mechanisms may combine to “affect health cumulatively over the life course and through multiple pathways, not only through their association with educational attainment or adult income” [37, p. 173].

Despite the impact of the Affordable Care Act that improved access to medical care, the racial health inequities studied in this paper are widening, rather than narrowing, especially among a distinct group of counties that have in common the resegregation of their schools in the 1990s. These widening disparities appear to be a legacy of intentional decisions made decades ago to reverse the gains stemming from court rulings based on *Brown v. Board of Education* [68]. Our finding that large increases in school segregation occurred between 1991 and 2000 highlights the fact that this period marked a broad retreat from integration efforts inspired by *Brown* [68]. Other intentional decisions may include the legacies of redlining and ongoing projects of gentrification. The most profound implication of this study’s findings is that systemic racism, especially in the form of racial school segregation, may have long-term consequences for racial health disparities that endure for decades.

## Limitations

There are several important limitations of this paper. First, because of limitations in data availability, we were not able to include all counties in the analysis, which could limit the generalizability of our findings. This is particularly important for our findings regarding infant mortality (*N* = 199 counties), total homicide (*N* = 183 counties), and firearm homicide (*N* = 104 counties). Nevertheless, even the 104 counties captured 49% of the Black US population.

Second, because of the collinearity between school and residential segregation, it can be difficult to tease out the effects of one versus the other. In the regression analyses, we chose to include the segregation measures that were least correlated, and the highest variance inflation factor in any of the regressions was 2.76. This does not generally indicate a problem with multicollinearity. Nevertheless, our results should be interpreted with caution for this reason.

Third, the COVID-19 pandemic began late in the study period and could have affected health outcomes for the year 2000, the final year of the study. Existing literature suggests that COVID-19 may have exacerbated racial disparities in a number of health outcomes [71].

## Conclusion

Despite these limitations, this paper provides new evidence that school segregation is an independent predictor of racial health disparities and that reducing school segregation—even in the face of high residential segregation—could have a long-term impact on reducing racial health disparities. In spite of how the US collectively has improved in its technological advances over time and in longevity for many individuals, health disparities continue to persist. School segregation is a factor that plays an influential part in exacerbating health inequities throughout the course of one’s life. If left unaddressed, school segregation could negatively impact the lives of current youth and future generations.

For example, although this study did not assess the presence of vending machine accessibility in the schools, it may be worthy to explore the relationship between changes in school segregation and other outcomes such as school nutrition environments. Such studies could provide demonstrations of if and how the school nutrition environments may influence students’ eating habits, thus influencing their long-term health outcomes of reproductive health, cognitive reasoning when faced with conflict and avoiding violence, and overall chronic health. Furthermore, they may provide important information essential to school policy and enhance the health of all youth including at-risk youth. Our findings indicate the need for programs that center community elevation and equitable infrastructure moving forward. Programs should be inclusive of those at the school-aged level or at earlier stages of life.

## Supplementary Information

Below is the link to the electronic supplementary material.

Supplementary file1 (PDF 911 kb)

## Data Availability

The database of mortality rate ratios and segregation indices produced in this research project is available upon request from the lead author.

## Abbreviations

CDC: Centers for Disease Control and Prevention
ICPSR: Inter-university Consortium for Political and Social Research
IHME: The Institute for Health Metrics and Evaluation
LSDD: Longitudinal School Demographic Dataset

## Code availability

Code used to run the trajectory analysis is available upon request from the lead author.
