# Supplementary material for: Association Between Changes in Racial Residential and School Segregation and Trends in Racial Health Disparities, 2000–2020: A Life Course Perspective
Source: J Racial Ethn Health Disparities. 2024 Feb 29;12(2):1278–97. doi: 10.1007/s40615-024-01960-y (PMC11914365; doi:10.1007/s40615-024-01960-y)
Supplement: Supplementary file 1 — Supplementary file1 (PDF 911 kb) [file 40615_2024_1960_MOESM1_ESM.pdf]

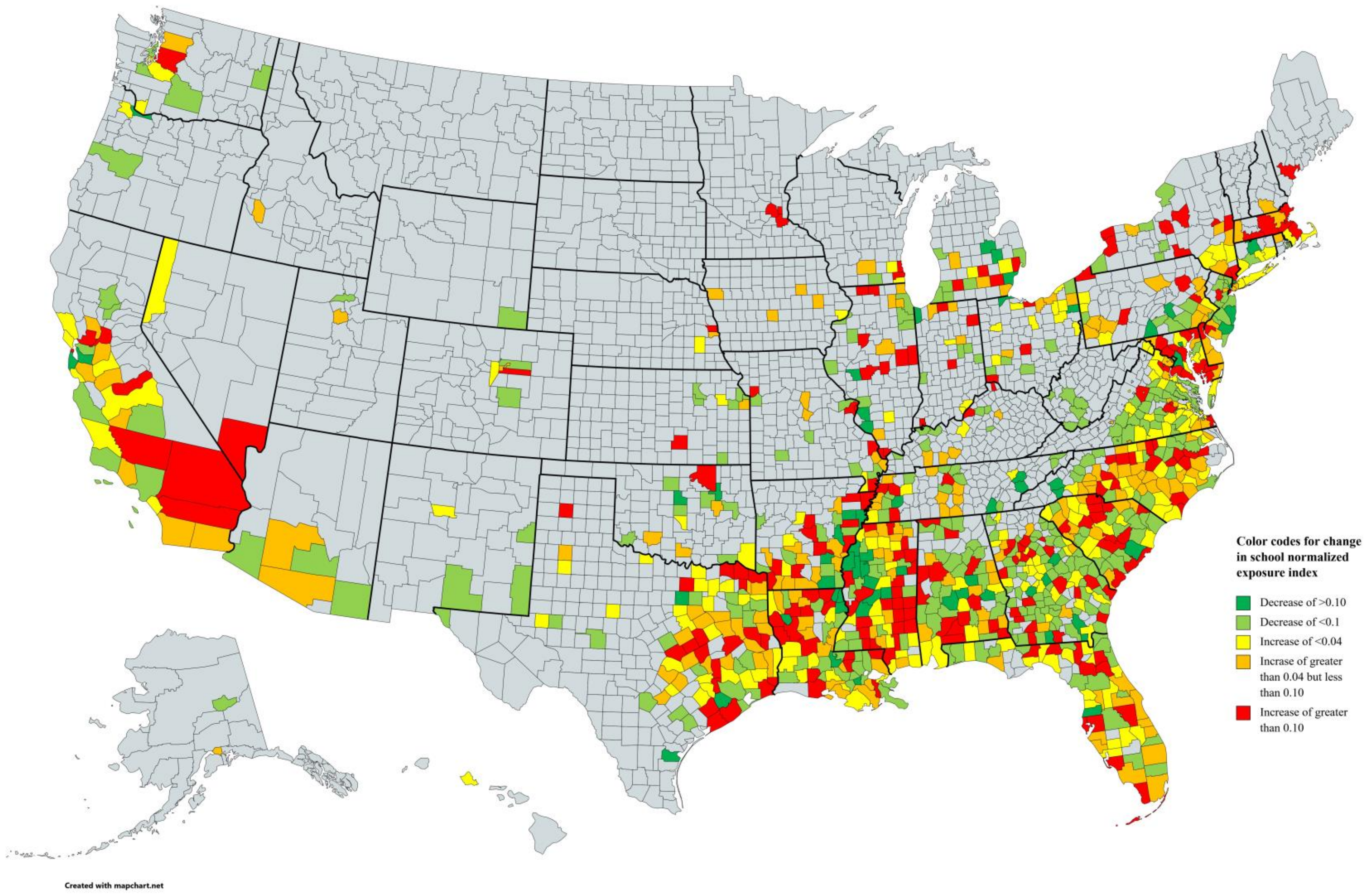

Supplemental Fig. 1 Heat Map Showing Change in Racial School Segregation (Normalized Exposure Index) from 1991 to 2000

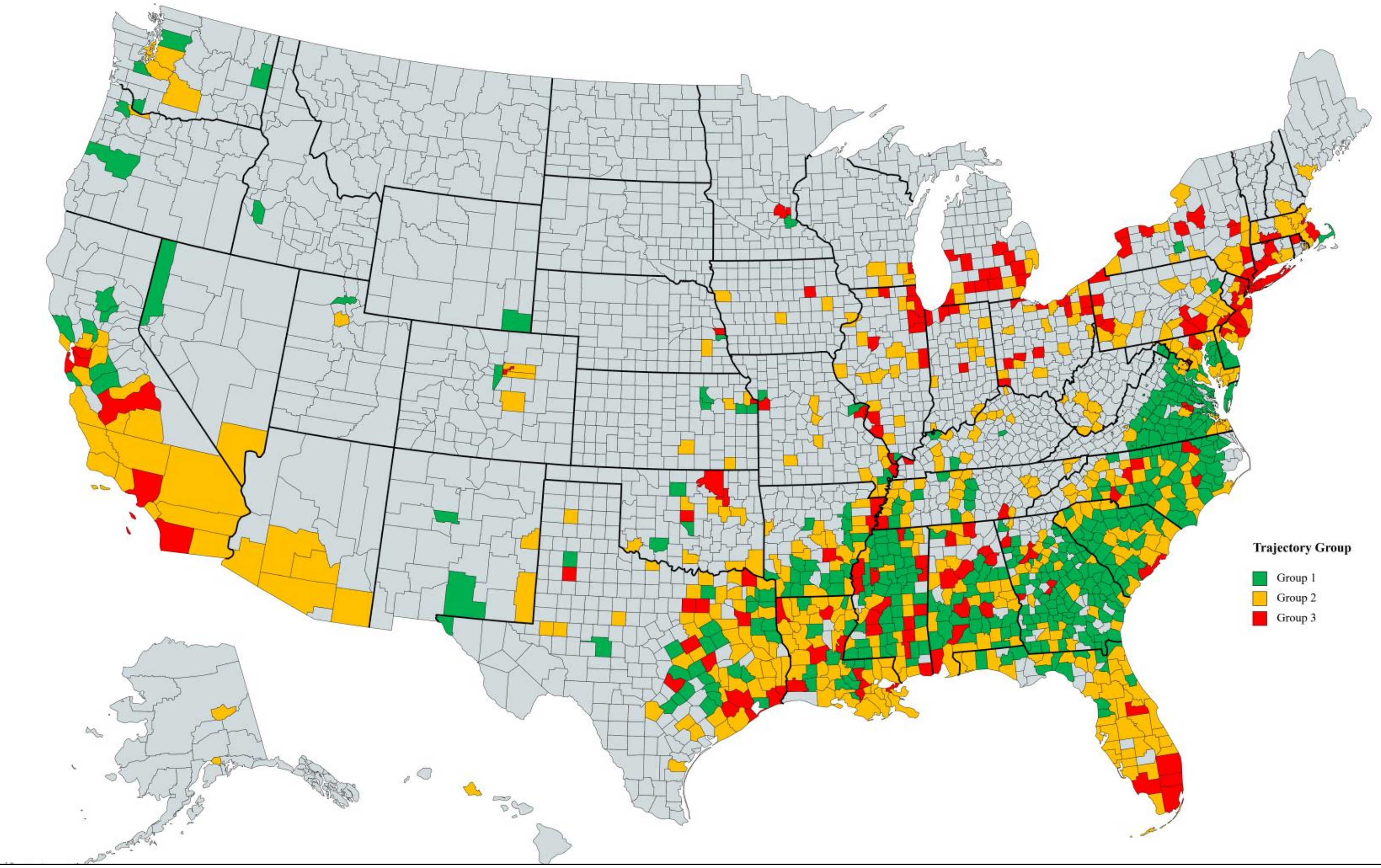

Supplemental Fig. 2 Heat Map Showing Which Counties are in Which Residential/School Multi-Trajectory Group
